# Quality of maternal healthcare in India: Has the National Rural Health Mission made a difference?

**Published:** 2011-06

**Authors:** Harish Nair, Rajmohan Panda

**Affiliations:** 1 Centre for Population Health Sciences and Global Health Academy, University of Edinburgh, Scotland, UK; 2 Public Health Foundation of India, New Delhi, India

## Abstract

Despite a five decade old Family Welfare programme, India still continues to contribute almost a quarter of the global estimates of maternal morbidity and mortality. Quality aspects in maternal health care have long been ignored in the Indian public health system. It is only with the launch of the National Rural Health Mission (NRHM) that quality of care has been accorded due recognition at the policy and planning levels of the national health programmes. Using review of available data sources and published literature, this paper aims to examine the scenario of quality of care in maternal health over the last decade and the impact of NRHM initiatives on the same. While NRHM has made efforts to address lacunae associated with quality of maternal care in the public health system, there is much scope for improvement.

The WHO estimates show that out of the 536 000 maternal deaths globally each year, 117 000 (22%) occur in India (1). In addition to these, millions suffer pregnancy related morbidity. According to Global Burden of Disease estimates for 2004, India contributes 21% of the disability adjusted life years (DALYs) lost due to maternal conditions (2). Public health initiatives over the last two to three decades have helped India to improve health indicators such as life expectancy and total fertility rate to a great extent, but some crucial indicators like Maternal Mortality Ratio (MMR) and Infant Mortality Rate (IMR) have stagnated at around 400 per 100 000 live births and 60 per 1000 live births, respectively, in the 1990s (3). Despite a series of national level safe motherhood policies and programmatic initiatives over the past two decades there is little evidence that maternity has become significantly safer in India. The National Rural Health Mission (NRHM) was launched with much fanfare in April 2005 “to provide accessible, affordable and quality health care to the rural sections especially the vulnerable populations” (4). An integral component of NRHM is the safe motherhood intervention in the form of Janani Suraksha Yojana (JSY) for reducing maternal and neo-natal mortality. JSY is a 100% centrally sponsored scheme under the umbrella of NRHM which integrates cash assistance with antenatal care during the pregnancy period, institutional care during delivery and immediate post-partum period in a health centre by establishing a system of coordinated care by field level health worker. Though the scheme has been successful in pushing up the institutional delivery rate in some high focus states, the ambitious goals of reducing the MMR from existing ratio of 301 to 100 per 100 000 live births, by 2012 (4) will not be possible if ‘quality’ aspects are ignored while addressing issues related to equity and access to health care for the Indian population. Addressing the issues of quality in maternal health service delivery is important not just to decrease the MMR and reduce maternal morbidity but also to instill confidence in the public health system amongst end users and thereby increase the demand for institutional deliveries. This alone will ensure that the gains made in the JSY scheme in the last 4 years will lead to the final expected outcome of the NRHM of decreasing maternal mortality and morbidity.

## QUALITY OF CARE: THE CONCEPT

The concept of quality of care is complex and multidimensional. The definition of quality of care is highly varied- ranging from excellence (5) to expectations or goals which have been met (6,7) to “degree to which health services for individuals and populations increase the likelihood of desired health outcomes and are consistent with current professional knowledge” (8). At a population level, quality of care can be defined as “ability to access effective care on an efficient and equitable basis for the optimisation of health benefit/well-being for the whole population” (9). All dimensions of quality of care reduce to two questions. First, can an individual get the care they need when they need it (ie, is the care accessible)? Second, when they get care, is it effective both in terms of clinical effectiveness and interpersonal relationships? This definition of quality of care is appropriate when applied at an individual level. This paper will largely restrict to analysing the quality of care in maternal health at the individual level through an equity lens.

Though India’s Health and Family Welfare Programme has been in existence for almost five decades, it is characterised by modest achievement and unfulfilled promise. Information on the services at the provider-client level remains limited, much of the evidence having become available only in the last decade with a good deal being unpublished and inaccessible to those interested in this issue (10).

### Access to care

**Figure 1:**
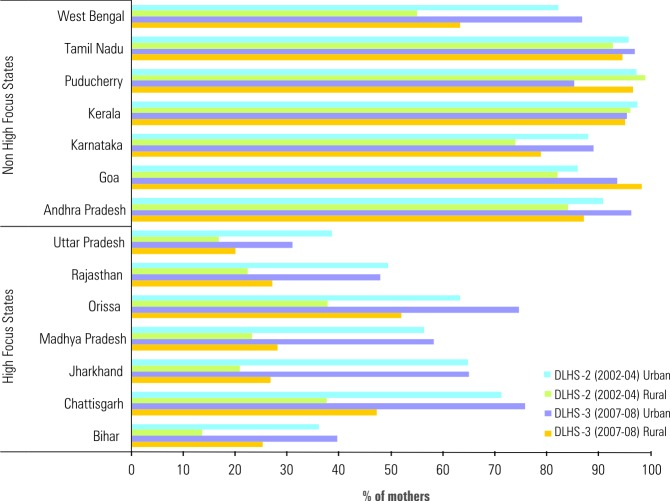
Comparative proportion of mothers who had three or more antenatal check ups during their last pregnancy. (DLHS-3 against DLHS-2). DLHS: District level health survey. High focus states under NRHM were 18 states identified for special attention based on weak public health indicators and/or weak health infrastructure.

Access to care is a vital but complex element of quality of care since it determines whether a client even gets to the service provider. The available community based evidence suggests that there is considerable variation in the level of outreach visits by the Auxiliary Nurse Midwife (ANM), largely by geographical location, with significantly higher visits in the southern and western than in the north Indian states. In a four state study conducted over a decade ago, 89% and 93% women surveyed in Tamil Nadu and Karnataka reported having been visited by a female paramedical worker in the last three months, compared with 53% and 61% women from Bihar and West Bengal, respectively (11). There were also differentials in access to care between urban and rural areas, if utilization of care is taken as a proxy for access to care. The National Family Health Survey-3 (NFHS-3) conducted during 2005-06 reports that only 62.4% of ever married women respondents living in urban areas reported having received the WHO recommended four antenatal visits compared to 27.7% rural women (12). The District Level Household Survey-3 (DLHS-3) conducted during 2007–2008 (13) indicates an overall improvement in access to maternal care (if three or more ante-natal check ups are taken as proxy) in the post NRHM period, perhaps more for the high focus states (with poor health indicators) than the non high focus states (which hitherto had better health indicators) (**Figure 1**).

**Figure 2:**
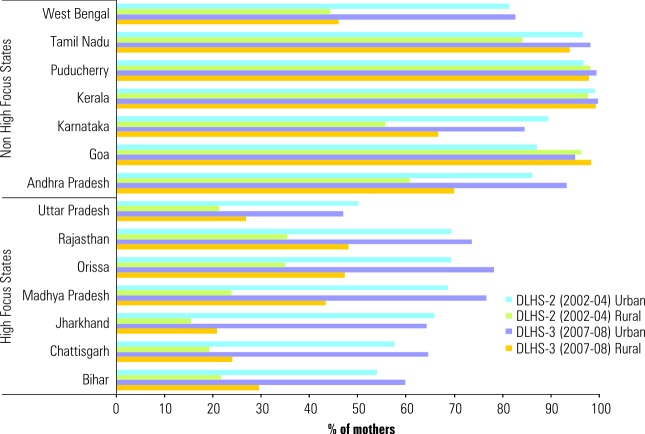
Comparative proportion of mothers who had safe delivery (ie, institutional delivery or home delivery attended by a skilled health personnel like doctor, nurse, LHV, ANM, midwife etc.) during last pregnancy. (DLHS-3 against DLHS-2). DLHS: District level health survey; LHV: Lady health visitor; ANM: Auxiliary nurse midwife. High focus states under NRHM were 18 states identified for special attention based on weak public health indicators and/or weak health infrastructure.

Data over the last three decades reveal that significant differences in frequency of outreach visit exist even within the same geographic region. One study from rural Maharashtra found that respondents residing in villages more remote from those, to which the ANM was assigned, were significantly less likely to have reported a recent visit by a health worker, to have been visited for meaningful lengths of time and to have received other maternal and child health services (14). An earlier study also found a much greater tendency for workers to visit communities and households accessible to main roads (15). NRHM does not appear to have made much of a difference in this regard. In Orissa, JSY beneficiaries had to travel, on average, 15.8 km to reach the ultimate place of delivery (16). Without an efficient referral system, women with complications are referred from facility to facility before they finally reach their place of delivery. This results in loss of precious time and contributes to one of the major delays responsible for maternal mortality. A study conducted in Andhra Pradesh showed that among the 98 women who used hospital facilities nearly sixty percent went to two or more hospitals. One woman had visited as many as nine hospitals and finally died at home (17). According to NFHS-3, more than half the births in India take place at the woman’s own home and 9% at parent’s home (12). Overall, only 47% of all deliveries are attended by a skilled birth attendant (SBA); 73.4% in urban areas compared to 37.4% in rural areas. The DLHS-3 data reveal that the rural-urban gap for safe deliveries remains wide as ever in the northern Indian states (13) (**Figure 2**).

Investigators in a study conducted at the beginning of the millennium and involving rural and urban women in Maharashtra have listed safety and good quality of care as one of the motivating factors for choosing to give birth at home (18). “In government hospital delivery room is not there.

Toilet and water facilities are not there in public health centre properly. So I felt safe to give birth in house,” remarked one of the respondents from Pune (18).

The Government of India constituted Common Review Missions (CRMs) under the NRHM to review the implementation of NRHM. The teams constitute of central and state government officials, public health professionals from the academia, public health activists from civil society organizations and representatives from development partners. The teams constituted for the Second Common Review Mission (CRM) (November – December 2008) reported that although there is some improvement in the levels of cleanliness and provision of waiting space for patients in the post 2005 period, cleanliness of toilets was still lacking (19). Assessments carried out on health facilities across India indicate a suboptimal degree of purchases, maintenance and utilization of general medical equipment and a lack of support facilities like 24-hour water and electricity supply (20). This is reinforced by the observations of one of the visiting State teams of the CRM.

“The infrastructure is old and requires repairs. OPD patient load is very high, institutional delivery load is also very high, however the PHC has only 4 beds which require to be augmented, there is no referral transport service available and laboratory services are inadequate” (19).

The findings of the Third Common Review Mission teams to Bihar, Chattisgarh and West Bengal in November 2009 indicate that very little has changed in the one year since the second CRM (21). The team visiting Bihar observed that the “basic utilities (toilet and running water) in the observed facilities were very poor and are not conducive for the women to stay for long after delivery” (22). Thus, insufficient public health care infrastructure, unclear accountability, and the lack of empathy towards the poor have severely limited the optimal reach of even available maternal health services in the public health system in India.

**Figure 3:**
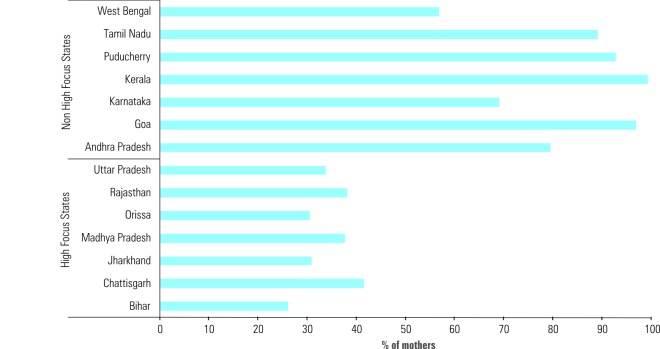
Proportion of mothers who received post natal care within 2 weeks of delivery during their last pregnancy. Data are overall for the state based on DLHS-3 (2007-2008). DLHS, District level health survey. High focus states under NRHM were 18 states identified for special attention based on weak public health indicators and/or weak health infrastructure.

Postnatal care is one of the most neglected components of maternal care. Data from NFHS-3 reveal that only 42% of women surveyed received postnatal care after their most recent delivery. Births to urban mothers are twice as likely to be followed by a postnatal check-up (66%) compared to their rural counterparts (34%) (12). The findings of DLHS-3 are no different – the rural-urban differential remains as wide as ever in the high-focus states (13) (**Figure 3**). It is thus evident that rural India where about 70% of Indian population resides has less accessibility to good quality care. Even in urban areas, lack of knowledge and awareness about health facilities among the poor, weak linkages between service providers and communities, and the limited role of community negotiating capacities severely impede the demand for health care services in these areas (23). Recent evaluation of the JSY in Orissa revealed that that only half of the JSY beneficiaries were given referral slips by Accredited Social Health Activist (ASHA) or other health personnel to help them access delivery services. The same report also notes: “With manifold increase in the institutional deliveries, quality of care has become an issue, for instance, women were discharged on average, 16 hours after normal delivery and there were instances of being discharged even within 3–4 hours after delivery. This is risky to the life of both mother and the newborn and would not serve the purpose of reducing maternal and neonatal mortality” (16).

Even the third CRM report indicates that mothers tend to be in institutions less than a day in most cases and that quality of care needs to improve in a large proportion of the health facilities (24).

### Clinical effectiveness

**Figure 4:**
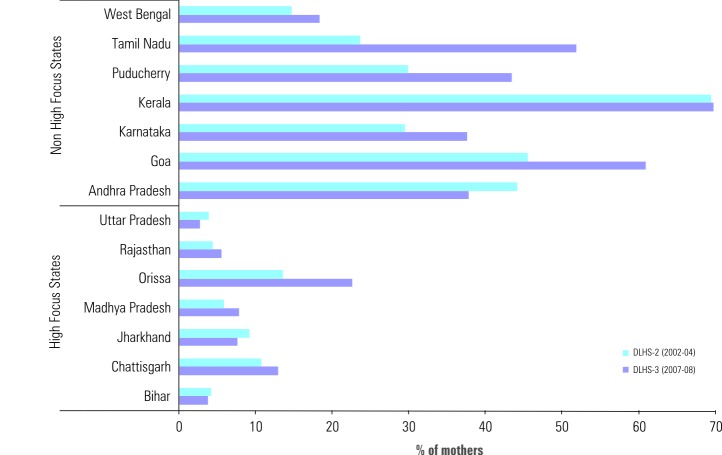
Proportion of mothers who had full antenatal care (at least 3 visits for ANC, one injection of tetanus toxoid and 100 tablets or equivalent thereof of iron and folic acid supplement) during their last pregnancy. Data are overall for the state based on DLHS-3 (2007– 2008). ANC: Antenatal care; DLHS: District level health survey. High focus states under NRHM were 18 states identified for special attention based on weak public health indicators and/or weak health infrastructure.

Khan and colleagues in a study from Bihar reported that 41% of the respondents felt that the time the health worker spent with them was very short and only 31% were fully satisfied with the visits they received (25). In another study from Maharashtra, almost two-thirds of the respondents reported that the ANM had spent less than five minutes in her most recent household visit (14). This lack of time spent by the ANM reflected on the lack of clinical effectiveness for those who manage to gain access to the care provided by the public health system. NFHS-3 data indicate that overall, only 15% women receive all recommended types of antenatal care, there being wide disparities between the states (4% in Uttar Pradesh compared to 64% in Kerala) (12). Though the DLHS-3 data indicate an overall improvement in clinical effectiveness of maternal health care (if full ante-natal check up which includes at least three ante-natal visits, one tetanus toxoid injection, 100 tablets of iron-folic acid supplement or its equivalent in syrup is taken as proxy), they appear to suggest that the improvement has been more in the non-high focus states which hitherto had better health indicators (13). In fact, some high focus states (e.g. Uttar Pradesh, Bihar and Jharkhand) appear to have deteriorated in the post-NRHM period (**Figure 4**).

**Figure 5:**
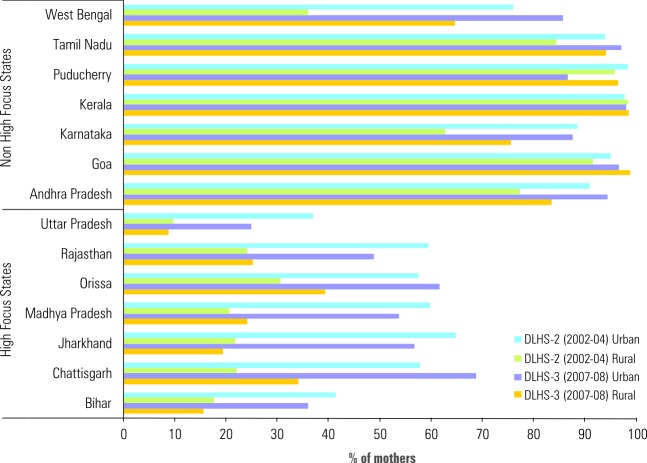
Comparative proportion of mothers who had their blood pressure measured at least once as part of antenatal care during their last pregnancy (DLHS-3 against DLHS-2). DLHS, District level health survey. High focus states under NRHM were 18 states identified for special attention based on weak public health indicators and/or weak health infrastructure.

Rani and colleagues have highlighted the north-south differential in a recent study involving secondary analysis of NFHS-2 data from four south Indian states (Andhra Pradesh, Karnataka, Kerala and Tamil Nadu) and four north Indian states (Bihar, Madhya Pradesh, Rajasthan, Uttar Pradesh) (26). The study shows that only 40.3% of the women receiving antenatal care in the north reported having their blood pressure measured during antenatal visit compared to 87.4% in the south. Though the DLHS-3 data indicate that these differentials persist, they present a greater cause for concern (13). In the post NRHM period, the northern Indian states of Bihar, Jharkhand, Madhya Pradesh, Rajasthan and Uttar Praesh have slipped further on this index of quality of care. (**Figure 5**). NFHS-3 reports that while 80–82% of the urban women had their blood pressure measured and weight taken, only 55% of the rural respondents reported having received these basic prerequisites of quality antenatal care (12). DLHS-3 data seem to indicate that the rural- urban differential has only grown wider in the post-NRHM period.

It is important to note that while southern states have made significant progress to address accessibility to good quality of care for antenatal care (ANC), they too fall woefully short of the standards in quality required to bring down maternal mortality. NFHS-3 indicates that 65% of the pregnant women received IFA during ANC which is a seven percentage point increase from NFHS-2. However, the survey also shows that that 59% of the pregnant women were found to be anaemic which is suggestive of poor quality of ANC resulting in poor compliance.

### Client – provider inter-personal relationships

Inter-personal relationship between the provider and the client is the key to improved client satisfaction, continued and sustained use of services and thereby better health outcomes. Government health clinics have long been accused of being apathetic and ignorant to client perspectives. It is thus no wonder that clients perceive private sector health services to be superior to that offered by the government program (14). The study on north-south differential cited earlier found that women in both the north and south India reported better quality of interpersonal care in the private sector (26). Births in a private facility are more likely to have a postnatal check-up at 6 weeks (85%) as well as a check-up within four hours of delivery (62%) than births in a public facility (76% and 53%, respectively) (12). Ravindran points out that the clients have a negative impression of government health facilities citing staffs’ and nurses’ verbal abuse of clients and demands for informal payments even for the most basic health services (27). A focus group study in Uttar Pradesh which documented perceptions among female respondents revealed that staff and medical officers in government institutions are often rude and discourteous to clients (28). Rao narrates the plight of an urban slum dweller in Bangalore who was slapped repeatedly by the nurses in a government hospital because she was too weak to bear down (29). “My mother’s house where I had my firstborn was better,” says the respondent. To add insult to the injury the hospital staff refused to hand over her baby until she made informal payments. Once she paid up, she was sent home within 24 hours of delivery without any medicines or postnatal check up.

Quality issues notwithstanding, government clinics continue to be used in large numbers because the costs to the clients are minimal. However, some studies are already revealing new evidences that the poor have also preferred to use the much costlier services provided by the largely unregulated private sector even when they have access to subsidized or free public health care (30). This is inherently regressive and has put a disproportionate burden for health care on poor households. It is not just the poor who face the double burden of poverty and ill-health, the financial burden of ill health can even push the non-poor into poverty.

The teams constituted under the second CRM in their final report re-iterated the need for attention to procedures for registration, patient flow and information through appropriate signage, waste disposal and other aspects crucial for a patient friendly facility (19). The shortage of human resources and thereby of the expected services was also noted as an issue of quality. The report of the Third CRM (unpublished) indicates that a positive outcome of the thrust of the changes that the NRHM has brought about in the last 3 years has been on infrastructure strengthening, facility improvement and enabling adequate numbers of human resources- and these measures seemed to have brought about a huge increase in institutional deliveries. It also concludes that even though “[t]he quality of care in the private sector is not necessarily much better than that reported for the public facilities, but because of the push of the system case loads seems to have migrated from the public system to the private system.” For example the team constituted under the Third CRM for the state of Gujarat observed that the quality of Chiranjeevi providers (a Public Private Partnership health provider scheme promoted by the State Government) is not necessarily better; however they are supported by a better demand generation involving the government workers at the village level and by a mindset that deems private sector provision better than government provision (31).The report submitted by the team recommends that increased patient load and overcrowding now at public health facilities can be resolved by planned efforts to rationalize patient load (deliveries) by upgrading the primary level services at Primary Health Centres and Subcentres. In general the report of the third CRM observes that lack of respect shown to the patients by the service providers is still a pervasive phenomenon that discourages use of public facilities.

## CONCLUSION

It is evident that quality is a more significant predictor of utilization of maternal health care than access. The second CRM report echoes the general finding in the high focus states that, “given the problems of the past, expectations of providers and even of the public had been set at very modest levels. The system is in danger of stabilizing at this low level of expectations and outputs, and even as one appreciates the effort that has gone in to reach this level, there is a need to set the benchmarks higher. There is much more that needs to be done, if the increased patient load and utilization of services was to manifest in increased outcomes” (19).

The report also recommends that improving the quality of care and comfort of stay for the in-patients in the public hospitals especially at the secondary level, through clean toilets, fresh linen, and a friendly environment are steps towards a system of ensuring quality improvement in all public health facilities. One of the major road blocks towards fostering a movement in enforcing quality of care in maternal health services has been the absence of independent advocates for promoting quality of care in this realm within the civil society. The third CRM report lays importance on the use of external assessment and certification of the facilities and for building a policy framework that mandates this.

In summary, although there has been some improvement in the quality of maternal health services in the last decade, India is still a long way off from the standards in most emerging economies leave alone developed countries. Unless the health system is able to ensure good quality care translating into continued and sustained use of maternal health services throughout the country, achievement of MDG-5 goal will likely remain out of reach for a long time.

## RECOMMENDATIONS

Though more NRHM has just completed five years of existence, scant data are available on the impact of the mission on quality of care in health facilities. It is imperative that further research is conducted to assess the impact of NRHM on maternal health services and the change it has brought about in client perspectives so that gains from the mission can be consolidated. Community based organisations and consumer groups will need to advocate for quality of care in maternal services by forging collaborations and sharing resources amongst all stakeholders involved in advocating for quality of care in maternal health services. This could be initiated by a pan national organisation which would be able to bring together national and international organisations like the White Ribbon Alliance, UNICEF, WHO, UNFPA and other international donors on a common platform. State Governments will need to establish task forces for enforcing Indian Public Health Standards (IPHS) guidelines at all levels and these should be monitored by an independent body at the centre. State Governments should also set up mechanisms for efficient procurement, management and monitoring of supply chain systems [on the lines of Tamil Nadu Medical Supplies Corporation (TNMSC)] for equipment and drugs for essential maternal health services. Standard treatment guidelines created in consultation with senior medical officials also need to be implemented and monitored. Hospitals need to be certified as women and baby friendly. Multi pronged strategies should also be worked out to improve the quality and efficiency of services being delivered by ASHAs as these will have a major impact on the success of NRHM in general and improvement of maternal health indicators in particular.
